# Association of birth order with adolescent mental health and suicide attempts: a population-based longitudinal study

**DOI:** 10.1007/s00787-018-1266-1

**Published:** 2019-01-02

**Authors:** Kayleigh E. Easey, Becky Mars, Rebecca Pearson, Jon Heron, David Gunnell

**Affiliations:** 1 School of Psychological Science, University of Bristol, Bristol, UK; 2 MRC Integrative Epidemiology Unit, University of Bristol, Bristol, UK; 3 Centre for Academic Mental Health, University of Bristol, Bristol, UK; 4 NIHR Biomedical Research Centre at the University Hospitals NHS Foundation Trust, Bristol, UK

**Keywords:** ALSPAC, Birth order, Suicidal behaviour, Mental health

## Abstract

Previous cohort studies have observed higher birth order to be associated with increased risk of suicidal behaviour. However, the mechanisms underlying this association are unclear. Using data from the Avon Longitudinal Study of Parents and Children (ALSPAC), we used multivariable logistic regression models and mediation analysis to investigate the associations of birth order with adolescent suicide attempts and psychiatric disorder. We investigated whether the number of maternal depressive episodes and father absence mediated the associations found. In fully adjusted models (*n* = 2571), higher birth order was associated with an increased risk of both suicide attempts (OR = 1.42, CI = 1.10–1.84) and psychiatric disorder (OR = 1.29, CI = 0.99–1.69). Maternal depression and father absence only partially mediated (8%; 12%) these associations. Whilst maternal depression and paternal absence partially mediated the associations between birth order, and suicidal behaviour and psychiatric disorder, other pathways may account for much of these associations. Future studies should investigate alternative mediating pathways.

**Electronic supplementary material:**

The online version of this article (10.1007/s00787-018-1266-1) contains supplementary material, which is available to authorized users.

## Introduction

Suicide is one of the most common causes of death among adolescents worldwide [28, 39], with self-harm and mental health problems shown to be the strongest risk factors for suicide attempts [20]. Previous studies have investigated factors which contribute to risk, and observed that later-born children have an increased risk of suicide [2] and hospital admission following a suicide attempt [24]. The association is seen in varying cultures [7] and using within-family designs, with each increase in birth order associated with a 18–46% increase in risk [2, 34].

The population attributable fraction (PAF) for the contribution of birth order as a risk factor for suicide is high (PAF = 0.24), theoretically suggesting that if we could remove the risk associated with later birth order, we could potentially prevent 24% of suicide deaths [32]. These findings highlight the need to further understand the mechanisms underlying such associations. There are several important considerations when studying associations between birth order and suicide/psychiatric disorders. These include the need to consider only children separately to those with siblings, as their circumstances may differ from those born to families with two or more children. Only children have been previously shown to have a greater risk of self-harm compared to second or third-born children [31], with previous research on birth order and suicide restricting investigation to families consisting of two or more children [2]. Maternal age must be considered also, as increased maternal age at delivery has been strongly associated with decreased suicide risk, with each 10 year increase in maternal age associated with a 57% reduction in risk [2]. In addition to birth order, family size (number of other siblings) also needs to be accounted for. Third-born children, for example, are by definition members of larger families and can add further issues of confounding if not controlled for.

Most existing studies investigating the association between birth order and suicide have been conducted in large population cohorts with limited adjustment for confounding factors (most commonly, gender, age and education variables) [2, 36]. Relatively few studies have investigated associations with non-fatal self-harm, and those that have focused only on hospital presenting cases, which account for less than 13% of self-harm episodes [19, 21]. There is also a lack of research investigating birth order and mental health disorders, and the findings from the existing literature are conflicting. For example, Putter et al. [30]. found middle children to be at a greater risk of depression. However, later studies with access to larger datasets such as registry data have found later-born children to be at a higher risk of mental health problems [3].

Several possible mechanisms underlying birth order associations have been suggested. These include a dilution of resources available for later-born children within larger families, split parental attention, and greater parental stress with higher parity [11, 12, 18, 29]. Prenatal effects have also been proposed of mothers with increased parity having depleted nutritional reserves, affecting foetal neurodevelopment [33]. Although various mechanisms have been proposed for how birth order might affect suicide risk, few studies have directly tested such pathways. Furthermore, there are also plausible mechanisms that have not been investigated. For example, the impact of parental mental health on offspring’s suicide risk has yet to be investigated within the context of birth order, despite evidence of parental depression having a negative effect on child development generally [22, 37], as well as associations shown for maternal depression and increased suicidal ideation in offspring [17]. Father absence during a child’s upbringing has also been associated with offspring’s depressive symptoms [9] and externalising problems [4] in adolescence. It is possible that the number of children may influence the marital relationship and potential breakdown. However, father absence has not been investigated as a possible mechanism underlying the association between birth-order effects and offspring’s mental health.

The current study utilised a population-based cohort study with self-reported data on suicide attempts and psychiatric disorder in adolescence. Our aims were to:

1. Explore the relationship between birth order, and later suicide attempts and psychiatric disorders.
2. Investigate possible mediators of these relationships, including maternal depression and father absence.

## Method

### Sample

The Avon Longitudinal Study of Parents and Children (ALSPAC) is an ongoing population-based study, which recruited pregnant women residing in Avon, UK with expected dates of delivery from 1st April 1991 to 31st December 1992. The core sample consisted of 14,541 pregnant women, of which 14,062 were live births and alive at 1 year of age. Participants have been regularly followed-up through clinic visits and questionnaires. Detailed information about ALSPAC is available on the study website which includes a fully searchable data dictionary of available data (http://www.bris.ac.uk/alspac/researchers/data-access/data-dictionary). For further details on the cohort profile, representativeness, and phases of recruitment, see Fraser et al. and Boyd et al. [6, 14]. Ethical approval for the study was obtained from the ALSPAC Ethics and Law Committee and the Local Research Ethics Committees.

**Fig. 1:**
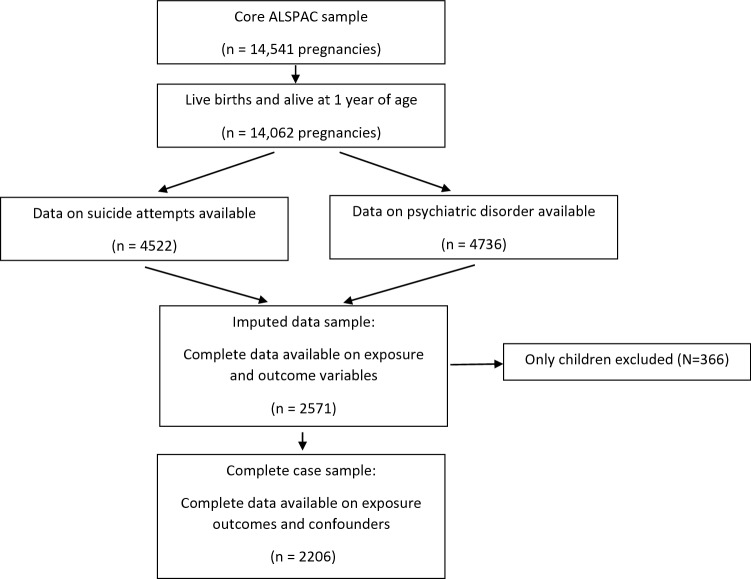
Offspring included in analysis of associations between birth order and suicide attempts/psychiatric disorder

4522 participants (59% female) from the ALSPAC cohort completed the self-harm questionnaire and were from families whose mother had not experienced a stillbirth/had a child die < 1 year of age. Of these, 2206 children had complete data for all variables used in the analyses and were from a family of 2 or more children (see Fig. 1).

### Measures

#### Outcome measures

##### Suicide attempts

The measure of suicide attempts was derived from questionnaires given to the ALSPAC participants at age 16 years. Those who responded positively to the question “Have you ever hurt yourself on purpose in anyway (e.g., by taking an overdose of pills or by cutting yourself)?” were classified as having a history of self-harm. Suicide attempts were classified as positive responses to the question “On any of the occasions when you have hurt yourself on purpose, have you ever seriously wanted to kill yourself?”, or by selecting the given option “I wanted to die” when asked “Do any of the following reasons help to explain why you hurt yourself on that (i.e. the most recent) occasion?”. Questions were based on those used in the Child and Adolescent Self-harm in Europe study [23].

##### Child’s psychiatric disorder

The Development and Wellbeing Assessment (DAWBA) [16] is a semi-structured interview about a child’s mental health symptoms and their impact. Diagnostic criteria for each disorder were given through a computer prediction according to International Classification of Diseases (ICD-10) or Diagnostic and Statistical Manual of Mental Disorders (DSM-IV), dependent on the mental health category. A binary measure was generated indicating the presence of any disorder (emotional or behavioural) on the DAWBA and used to assess a child’s psychiatric disorder at age of 15 years.

### Exposure variables

#### Birth order

Birth order was derived from a recorded measure of parity at 18 weeks gestation, here the mothers were asked the number of previous pregnancies resulting in either a livebirth or stillbirth. Within the current study, birth order was categorized as first born, second born and third plus born. Study children whose mothers had ever had a stillbirth (*n *= 115) or child who died within the first year of life (*n* = 174) were excluded from analyses.

#### Family size

At 47 months, the mothers were asked how many siblings (including half/step-siblings) the ALSPAC study child had living with them for at least 1 day a week. This variable was used to derive family size, and responses were categorized as one child, two children, or three or more children. Only children (without siblings) were removed before analysis (*n *=366).

#### Mediating variables

##### Maternal depressive episodes

The Edinburgh Postnatal Depression scale (EPDS) is validated for use in non-postnatal women [8] and was completed by the ALSPAC mothers at various time points. Maternal scores on the EPDS were totaled for complete measures of EPDS at 18 and 32 weeks gestation, 8 weeks, 8 months, 21 months, 33 months and 61 months post-birth.

##### Father absence

The mothers completed regular questionnaires asking whether the study child’s father still lived with the family (aged 1 year 7 months, 2 years 7 months, 3 years 9 months, 7 years, 8 years and 10 years). Responses were categorized as father present, father absent before age 5, and father absent aged 5 or over.

### Confounding variables

Confounding variables were chosen based on the previous literature that has investigated birth order and suicide attempts. We did not adjust for variables that could potentially lie on the causal pathway. The following variables were included as potential confounders in the analysis: mother’s social class (professional/managerial, or other) measured during pregnancy, income (divided into quintiles) measured at age 3 and 4 years, gestational age (length of pregnancy in weeks), maternal age (at delivery), maternal tobacco (if smoked in first 3 months of pregnancy) and alcohol (amount in first 3 months of pregnancy) consumption during pregnancy.

### Statistical analysis

Logistic regression analyses were used to investigate associations between birth order and the two outcome measures (suicide attempts and psychiatric disorder). The impact of confounders was explored by comparing unadjusted models to those adjusted for related confounding variables.

To disentangle the potential influences of birth order and family size, we estimated some preliminary logistic models for each of child’s suicide attempts and psychiatric disorder in turn. After excluding only children, we first estimated a model containing both birth order and family size as categorical independent variables as well as their (multiplicative) interaction. A series of more parsimonious models was then estimated, removing first the interaction and subsequently each main effect, using changes in log likelihood to monitor any detrimental change in model fit. Such analyses provide evidence of an independent effect of birth order, but not for family size which was not retained in either outcome model.

Primary analysis was conducted on imputed data. Multiple imputation by chained equations (MICE) in Stata [35] was used to generate 100 imputed datasets, and those with complete outcome and birth order data (*n *= 2571). This method assumes data are missing at random, whereby any systematic differences between the missing and the observed values can be explained by differences in observed data [38]. Several auxiliary variables available from the ALSPAC cohort were used to assist in the imputation. These included measures of the predictive factors used in the main analysis such as social class and other measures related to the outcomes, such as the EPDS and Mood and Feelings Questionnaire (MFQ) [1] collected at earlier ages.

### Mediation analyses

Separate mediation analyses were conducted using Mplus version 7 [26] to examine whether the number of maternal depressive episodes for the first 61 months of the study child’s life, and paternal absence during the child’s first 47 months mediated the association between birth order and suicide attempts/psychiatric disorders. In a final model, both potential mediators were tested in a single model to assess their combined affect.

The four assumptions necessary for mediation [25] are:

- No unmeasured confounding for exposure (X) to outcome (Y).
- No unmeasured confounding for X to mediator (M).
- No unmeasured confounding for M to Y.
- X must not cause any confounders for M to Y.

Mediation models were adjusted for confounders on all pathways X–M, M–Y, X–Y, in a first model adjusting for maternal age only and in an adjusted model using maternal age, social class, income, gestational age, alcohol and tobacco use in pregnancy. Results from path analyses conducted on a categorical outcome are given as probit regression coefficients.

All analyses were conducted using Stata version 14.1 and Mplus version 7.

## Results

Table 1 shows the distribution of study outcomes, stratified by birth order for the 2206 cohort members with complete data. Altogether, 128 (6%) participants indicated they had attempted suicide on at least one occasion, and 116 (5%) had a psychiatric disorder according to ICD-10 and DSM-IV diagnosis.

**Table 1:** Comparison of outcomes and potential categorical confounders, by birth order

	Birth order		
	First born	Second born	Third + born
Suicide attempts			
No	924 (95%)	838 (93%)	316 (93%)
Yes	44 (5%)	59 (7%)	25 (7%)
Any psychiatric disorder			
No	926 (96%)	846 (94%)	318 (93%)
Yes	42 (4%)	51 (6%)	23 (7%)
Socioeconomic position			
I–II	674 (70%)	595 (66%)	228 (67%)
III–V	294 (30%)	302 (34%)	113 (33%)
Income			
1st quintile (highest)	293 (30%)	234 (26%)	60 (18%)
2nd quintile	238 (25%)	221 (25%)	76 (22%)
3rd quintile	221 (23%)	180 (20%)	54 (16%)
4th quintile	140 (14%)	157 (17%)	95 (28%)
5th quintile (lowest)	76 (8%)	105 (12%)	56 (16%)
Smoked during pregnancy			
No	860 (89%)	807 (90%)	296 (87%)
Yes	108 (11%)	90 (10%)	45 (13%)
Drank alcohol during pregnancy			
No	473 (49%)	359 (40%)	116 (34%)
Yes	495 (51%)	538 (60%)	225 (66%)
Maternal age (years)			
15–25	227 (24%)	108 (12%)	11 (3%)
26–35	710 (73%)	705 (79%)	248 (73%)
36 +	31 (3%)	84 (9%)	82 (24%)

I–II: Professional and managerial occupations

III–V: Non-manual/manual/semi-skilled manual and unskilled manual

All further presented results are for analyses using the imputed dataset (*n *= 2571). Table 2 shows associations between birth order and the outcome variables suicide attempt, and psychiatric disorders. There was a graded increase in the odds ratio for both outcomes with increasing birth order. Controlling for a range of potential confounding variables had little effect on the associations observed. In the adjusted analyses, each unit change in birth order was linearly associated with an increased odds of suicide attempt [odds ratio (OR) = 1.42, (95% CI = 1.10–1.84)], and psychiatric disorder (OR = 1.29, 95% CI = 0.99–1.69). A dose–response effect was shown with an increase in suicide attempts for each increase in birth order, for second-born (OR = 1.56, 95% CI = 1.05–2.31) and third plus born children (OR = 1.97, 95% CI = 1.17–3.34).

**Table 2:** Association of birth order with suicide attempts and psychiatric disorders

	Suicide attempts				Psychiatric disorders			
	Unadjusted		Adjusted^a^		Unadjusted		Adjusted^a^	
	OR (CI)	*p*	OR (CI)	*p*	OR (CI)	*p*	OR (CI)	*p*
Birth order (*n *= 2571)								
First born	1.00	0.040^b^	1.00	0.022^b^	1.00	0.275^b^	1.00	0.137^b^
Second born	1.44 (0.99–2.10)		1.56 (1.05–2.31)		1.12 (0.76–1.64)		1.21 (0.81–1.80)	
Third + born	1.74 (1.10–2.76)		1.97 (1.17–3.34)		1.47 (0.92–2.36)		1.72 (1.01–2.94)	
Linear trend	1.33 (1.07–1.67)	0.012	1.42 (1.10–1.84)	0.006	1.20 (0.95–1.51)	0.129	1.29 (0.99–1.69)	0.056

^a^Adjusted for social class, income, maternal age at delivery, gestational age, alcohol consumption during months 1–3 of pregnancy, tobacco smoked during months 1–3 of pregnancy

^b^Wald test

Exploratory analyses showed that when maternal age was removed from the fully adjusted models, associations were attenuated for both suicide attempts (OR = 1.26, 95% CI = 1.01–1.58) and psychiatric disorders (OR = 1.14, 95% CI = 0.90–1.45).

The findings from the imputed data and complete case analyses did not differ substantially (see supplementary Tables 1, 2).

### Mediation models

**Fig. 2:**
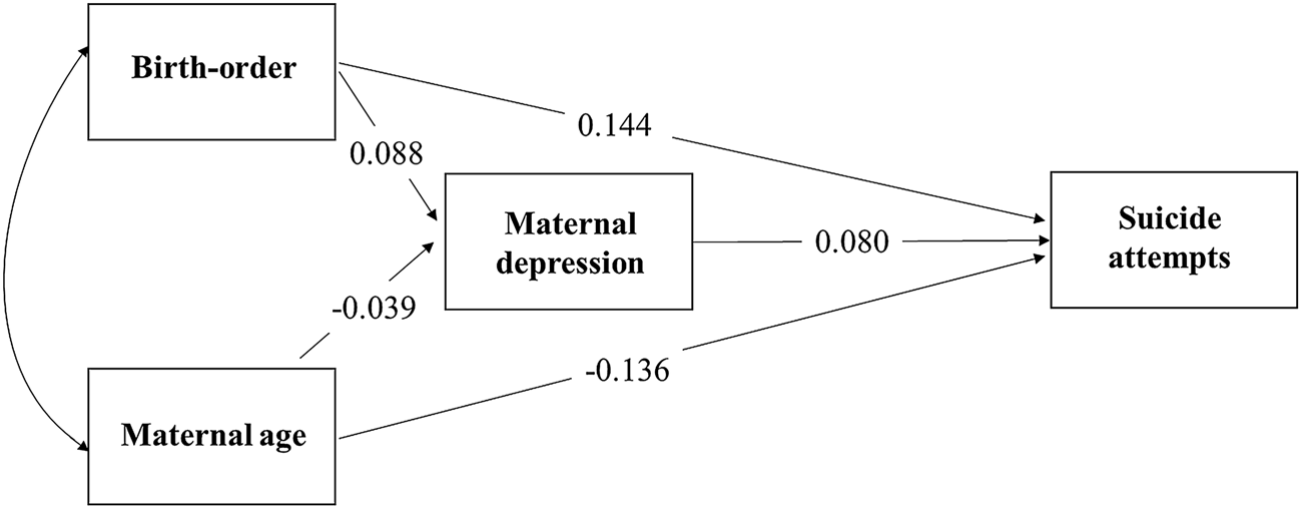
Path coefficients for mediation pathways to suicide attempts from birth order, adjusted for maternal age at delivery

Table 3 shows how the associations previously seen (in Table 2) for birth order and suicide attempts/psychiatric disorders are mediated by the number of maternal depressive episodes and father absence. The total effect shows the effect from all included variables within the model (exposure, mediators, outcome, and confounders). The indirect effect shows how much of the overall association between the exposure and outcome (e.g., birth order and suicide attempts) can be explained by each of the included mediators (maternal depression/father absence), as illustrated in Fig. 2.

**Table 3:** Mediation analyses exploring the number of maternal depressive episodes and father absence as mechanisms linking birth order with later suicide attempts and psychiatric disorders

*n *= 2571	Total effect		Maternal depressive episodes			Father absence			Maternal depressive episodes and father absence^c^		
	Total (SE)	*p*	Indirect *β* (SE)	*p*	% mediated	Indirect *β* (SE)	*p*	% mediated	Total indirect *β* (SE)	*p*	% mediated
Suicide attempts^a^	0.151 (0.041)	< 0.001	0.012 (0.005)	0.015	8	0.012 (0.005)	0.011	8	0.021 (0.006)	0.001	14
Suicide attempts^b^	0.120 (0.042)	0.005	0.006 (0.003)	0.086	5	0.006 (0.003)	0.062	5	0.010 (0.016)	0.006	8
Any psychiatric disorder^a^	0.107 (0.043)	0.012	0.016 (0.005)	0.003	15	0.008 (0.005)	0.104	8	0.020 (0.006)	0.002	19
Any psychiatric disorder^b^	0.090 (0.044)	0.039	0.008 (0.004)	0.033	9	0.005 (0.003)	0.179	6	0.011 (0.005)	0.021	12

^a^Adjusted for maternal age at delivery

^b^Adjusted for social class, income, maternal age at delivery, gestational age, alcohol consumption during 1–3 months of pregnancy, tobacco smoked during 1–3 months of pregnancy

^c^Mutually adjusted

Within the fully adjusted models, there was some evidence for a small indirect pathway through the number of maternal depressive episodes, from birth order to suicide attempts (*β *=0.006, SE = 0.003) and psychiatric disorders (*β *=0.008, SE = 0.004). As well as some evidence for a small indirect pathway through father absence, for suicide attempts (*β *=0.006, SE = 0.003) and psychiatric disorders (*β *=0.005, SE = 0.003). The strongest mediation effects were shown from the model of an indirect pathway through both the number of maternal depressive episodes and father absence, to suicide attempts (*β *=0.010, SE = 0.016, 8% of the total association) and psychiatric disorders (*β *= 0.011, SE = 0.005, 12% of the total association).

## Discussion

In this population study, we found higher birth order (later-born) children were at increased risk of suicide attempts and psychiatric disorders in adolescence. This is comparable to previous research investigating the effect of parity on offspring suicide attempts, which showed similar dose–response effects as the current study with an increase in suicide attempts shown for each increase in birth order [2, 34]. The pattern of association was similar for psychiatric disorders, although the statistical evidence of a dose–response effect was weaker. Few previous studies have investigated the association of birth order and offspring psychiatric disorder, the current study adds support to previous studies that have and that reported later-born children to be at greatest risk of mental health problems, and increased risk of suicide [3, 15, 31]. Any differences in strength of association for outcomes of suicide attempts and psychiatric disorder could reflect a different aetiology of suicidal behaviour [20] and mental health, and the relative contribution of birth order to these outcomes.

This study sought to investigate potential mechanisms underlying these associations and is the first to directly assess the effect of maternal depression and father absence as underlying mechanisms. Mediation analyses identified that the associations we found between increased birth order and both suicide attempts and psychiatric disorders, were partially mediated by the number of maternal depressive episodes and paternal absence. The strength of these mediation effects was small but does highlight a potentially modifiable pathway to risk.

Moving forward, it is important to investigate alternative pathways that contribute to the birth order associations; these might include bullying by older siblings and other socioeconomic indicators. Few studies have investigated the influence of sibling bullying on mental health problems, yet emerging studies have shown sibling bullying as a potential risk factor for mental health problems and self-harm [5, 10]. Future work investigating the prevalence of sibling bullying and as alternative pathway to suicide attempts is suggested.

Comparable to previous research [2], the mediation analyses in the current study found maternal age to be negatively associated with offspring suicide attempts, in that offspring born to older mothers had a decreased risk of suicide attempts. As the associations between birth order and suicide attempts, and maternal age and suicide attempts are in the opposite direction, maternal age likely suppresses the association with birth order, as shown in the adjusted models. Such findings suggest that greater support after birth may benefit younger mothers.

The current study has several notable strengths. These include using the ALSPAC cohort, a large, well-characterized cohort; with measures of a range of exposures recorded prospectively long before the study outcomes. Also, few studies have looked at suicide attempts in the community, which is important, as most young people who self-harm do not present to medical services [19, 20].

Potential limitations of the current study are the loss to follow-up, which may have led to selection bias, however, findings from the complete case and imputed data are similar suggesting this is unlikely. As noted above, other factors, such as sibling bullying and changes in the social and economic environment within the family, that were not investigated in this analysis, could contribute to the association with birth order. Of note however, studies using within-family designs have reported similar findings to ours [2, 34] and such designs should account for within-family socioeconomic circumstances, unless these affect offspring of different birth order in varying degrees. Another potential limitation is the use of self-reported measures of self-harm with suicidal intent. Adolescents may be ambivalent or fluctuate with regard to their intent to die, and reports may be influenced by the current mood state. The approach used in the current study was to classify participants as having attempted suicide if they reported any ‘non-zero’ level of suicidal intent, which is in line with previous research [27]. Self-harm could also have been under-reported by participants, however, this is less likely to have occurred due to the use of self-report questionnaires instead of interview-based reports [13].

The findings from this study provide further evidence that later-born children are at greater risk for suicide attempts and mental health problems. This is the first study to include postnatal maternal depression and father absence as potential mediators for this association. As number of maternal depressive episodes and father absence were shown to mediate some of these effects, family interventions for multiparous parents particularly for those with three or more children, may provide support for managing stress and partner relationships.

## Electronic supplementary material

Below is the link to the electronic supplementary material.

Supplementary material 1 (DOCX 21 kb)
