# Supplementary material for: Association of birth order with adolescent mental health and suicide attempts: a population-based longitudinal study
Source: Eur Child Adolesc Psychiatry. 2019 Jan 2;28(8):1079–86. doi: 10.1007/s00787-018-1266-1 (PMC6675759; doi:10.1007/s00787-018-1266-1)
Supplement: Supplementary file 1 — Supplementary material 1 (DOCX 21 kb) [file 787_2018_1266_MOESM1_ESM.docx]

| Table S1: Association of birth-order with suicide attempts and psychiatric disorders, for complete case data | | | | | | | | | |
| --- | --- | --- | --- | --- | --- | --- | --- | --- | --- |
|  |  | Suicide attempts | | | | Mental health disorder | | | |
|  |  | Unadjusted | | Adjusted^1^ | | Unadjusted | | Adjusted^1^ | |
|  |  | OR (CI) | *p* | OR (CI) | *p* | OR (CI) | *p* | OR (CI) | *p* |
| Birth-order  *n* = 2206 | First born | 1.00 | 0.070 | 1.00 | 0.075 | 1.00 | 0.178 |  | 0.097 |
|  | Second born | 1.48 (0.99-2.21) |  | 1.53 (1.01-2.32) |  | 1.33 (0.87-2.02) |  | 1.42 (0.92-2.20) |  |
|  | Third + born | 1.66 (1.00-2.76) |  | 1.74 (0.99-3.08) |  | 1.59 (0.94-2.69) |  | 1.85 (1.03-3.33) |  |
| Linear trend |  | 1.31 (1.03-1.67) | 0.028 | 1.35 (1.03-1.77) | 0.032 | 1.27 (0.99-1.64) | 0.064 | 1.37 (1.03-1.82) | 0.031 |

^1^Adjusted for social class, income, birthweight, maternal age at delivery, gestational age, alcohol consumption during months 1-3 of pregnancy, tobacco smoked during months 1-3 of pregnancy. ^2^Omnibus *p*-values

| Table S2: Mediation analysis for maternal depression and father absence to suicide attempts and psychiatric disorders, for complete case data | | | | | | | | | | | |
| --- | --- | --- | --- | --- | --- | --- | --- | --- | --- | --- | --- |
| *n*=2206 | Total effect | | Maternal depression as mediator | | | Father absence as mediator | | | Maternal depression and father absence combined | | |
|  | Total (SE) | *p* | Indirect *β* (SE), CI | *p* | % mediated | Indirect *β* (SE), CI | *p* | % mediated | Total indirect *β* (SE) | *p* | % mediated |
| Suicide attempts^1^ | 0.133 (0.044) | 0.003 | 0.009 (0.005) | 0.037 | 7 | 0.013 (0.005) | 0.019 | 10 | 0.019 (0.006) | 0.003 | 14 |
| Suicide attempts^2^ | 0.102 (0.046) | 0.027 | 0.004 (0.003) | 0.166 | 4 | 0.008 (0.004) | 0.049 | 7 | 0.011 (0.005) | 0.023 | 11 |
| Any psychiatric disorder^1^ | 0.123 (0.046) | 0.007 | 0.013 (0.005) | 0.007 | 11 | 0.008 (0.006) | 0.174 | 7 | 0.018 (0.007) | 0.010 | 15 |
| Any psychiatric disorder^2^ | 0.109 (0.047) | 0.021 | 0.007 (0.004) | 0.089 | 6 | 0.005 (0.004) | 0.240 | 5 | 0.010 (0.006) | 0.075 | 9 |
| ^1^ Adjusted for maternal age.  ^2^: adjusted by ^2^ Adjusted for social class, income, maternal age at delivery, gestational age, alcohol consumption during 1-3 months of pregnancy, tobacco smoked during 1-3 months of pregnancy. | | | | | | | | | | | |
